# Impact on Quality of Documentation and Workload of the Introduction of a National Information Standard for Tumor Board Reporting

**DOI:** 10.1200/CCI.19.00050

**Published:** 2020-04-23

**Authors:** Kees C. W. J. Ebben, Melle S. Sieswerda, Ernest J. T. Luiten, Joan B. Heijns, Carmen C. van der Pol, Maud Bessems, Aafke H. Honkoop, Mathijs P. Hendriks, Janneke Verloop, Xander A. A. M. Verbeek

**Affiliations:** ^1^ Department of Research and Development, Netherlands Comprehensive Cancer Organization, Utrecht, the Netherlands; ^2^ Department of Surgical Oncology, Amphia Hospital, Breda, the Netherlands; ^3^ Department of Medical Oncology, Amphia Hospital, Breda, the Netherlands; ^4^ Department of Surgical Oncology, Alrijne Hospital, Leiden, the Netherlands; ^5^ Department of Surgical Oncology, Jeroen Bosch Hospital, ’s-Hertogenbosch, the Netherlands; ^6^ National Breast Cancer Network Netherlands (NABON), Utrecht, the Netherlands; ^7^ Department of Medical Oncology, Isala Hospital, Zwolle, the Netherlands; ^8^ Department of Medical Oncology, Northwest Clinics, Alkmaar, the Netherlands

## Abstract

**PURPOSE:**

Tumor boards, clinical practice guidelines, and cancer registries are intertwined cancer care quality instruments. Standardized structured reporting has been proposed as a solution to improve clinical documentation, while facilitating data reuse for secondary purposes. This study describes the implementation and evaluation of a national standard for tumor board reporting for breast cancer on the basis of the clinical practice guideline and the potential for reusing clinical data for the Netherlands Cancer Registry (NCR).

**METHODS:**

Previously, a national information standard for breast cancer was derived from the corresponding Dutch clinical practice guideline. Using data items from the information standard, we developed three different tumor board forms: preoperative, postoperative, and postneoadjuvant-postoperative. The forms were implemented in Amphia Hospital’s electronic health record. Quality of clinical documentation and workload before and after implementation were compared.

**RESULTS:**

Both draft and final tumor board reports were collected from 27 and 31 patients in baseline and effect measurements, respectively. Completeness of final reports increased from 39.5% to 45.4% (*P* = .04). The workload for tumor board preparation and discussion did not change significantly. Standardized tumor board reports included 50% (61/122) of the data items carried in the NCR. An automated process was developed to upload information captured in tumor board reports to the NCR database.

**CONCLUSION:**

This study shows implementation of a national standard for tumor board reports improves quality of clinical documentation, without increasing clinical workload. Simultaneously, our work enables data reuse for secondary purposes like cancer registration.

## INTRODUCTION

Tumor boards,^1^ clinical practice guidelines,^2^ and cancer registries^3^ are intertwined cancer care quality instruments. Tumor boards perform two separate tasks. First, they perform a multidisciplinary review of the patient status, during which data previously reported by ancillary services (eg, radiology or pathology) may be aggregated or reinterpreted.^4-7^ For example, a tumor board may decide that, for a particular case, a tumor diameter is better approximated on ultrasound than on magnetic resonance imaging, which may lead to readjustment of the tumor stage. Subsequently, on the basis of the outcome of the review, the tumor board will recommend a course of action. This final recommendation, together with any (potentially readjusted) findings that drive it, should be documented in the electronic health record (EHR) in a tumor board report.^8^

Clinical practice guidelines are the embodiment of the current status of scientific knowledge and (should) form the basis of the tumor board recommendations.^2^ However, the format of most guidelines is far from ideal for consultation at the point of (multidisciplinary) decision making.^9^

Cancer registries, such as the Netherlands Cancer Registry (NCR), collect patient data generated during routine care. They are the basis for epidemiologic outcomes research, results of which are used to evaluate and refine the guidelines used in tumor board decision making.

In the current situation, there are several areas of improvement: tumor board report quality, clinician workload, and data reuse. First, quality of tumor board reports varies widely between hospitals, while at the same time it is easily understood that proper documentation may directly influence patient outcomes.^10^

Second, although valuable for patient care, tumor board meetings place a burden on physicians’ time. Because of the meeting’s quick pace, preparation is required, which consists of summarizing patient history, clinical findings, and ancillary information. This is complex work: it requires knowledge and skill to reconstruct a patient’s medical timeline from progress notes and to determine what is relevant for the upcoming discussion. After the meeting, it is customary to notify the patient’s general practitioner (GP) of the outcome by means of a clinical letter. This too places a burden on time.^11^

**CONTEXT**

**Key Objective**
To develop a national standard for a structured tumor board report for breast cancer and implement this in an electronic health record and work processes in a Dutch hospital. This was followed by a clinical evaluation of the impact on quality of the tumor board report, workload, and interoperability (ie, the degree to which stakeholders are able to electronically exchange information).
**Knowledge Generated**
This study shows that standardized structured tumor board reporting can successfully be implemented in complex multidisciplinary processes. The adherence and implementation of a national standard benefits quality of tumor board reporting but also improves interoperability in cancer care without significant additional workload.
**Relevance**
Improving interoperability among stakeholders in health care is an important topic. The development and implementation of standards is seen as a prerequisite for interoperability. This study proposes and evaluates a generalizable approach to standard development that is also applicable beyond breast cancer and even oncology.

Third, cancer registries to date either rely on self-reporting by hospitals or use professionally trained data managers (cancer registrars) to obtain data from medical records.^12^ In both cases, this requires significant effort.

Standardized structured reporting has been proposed as a solution to support clinicians to produce more complete and consistent documentation.^13,14^ It entails capturing data in discrete fields using (international) terminology systems, like Systematized Nomenclature of Medicine (SNOMED). As a result, reuse of information for secondary purposes is facilitated at the same time.^15,16^

In this study, we describe the implementation and evaluation of a national standard for tumor board reporting for breast cancer care on the basis of the national guideline affected, according to the method described by Hendriks et al.^17^ We investigated the effects of the implementation regarding the quality of clinical documentation, with the associated workload, and reuse of data for the NCR and automatic text generation for GP letters. In addition, we investigated the effect on additional data entry and changes required during the tumor board meeting and whether cancer registrars can play a role in supporting tumor board preparation.

## METHODS

### Design

For this study, a before-after design was used. Data collection took place before (baseline measurement) and after (effect measurement) implementing the national standard for breast cancer tumor board forms (forms are defined as predefined questionnaires). The study design was submitted to a medical ethics committee but was considered exempt from approval.

### Definition of the National Information Standard

Previously, we derived a national information standard for breast cancer from the corresponding Dutch clinical practice guideline. Briefly, the guideline was analyzed and translated into clinical decision trees.^17^ In a decision tree, nodes, branches, and leaves represent data items (patient or disease characteristics; eg, tumor diameter), values or cutoff points (eg, ≤ 5 mm), and guideline recommendations (eg, perform a lumpectomy), respectively. We encoded the data items, together with any value sets, using international standards (eg, SNOMED) where possible. The resulting list of data items makes up the information standard for breast cancer.

The information standard was approved by the EHR standardization workgroup of the National Breast Cancer Network of the Netherlands, members of which are surgeons, physicians, radiation oncologists, radiologists, pathologists, clinical geneticists, nuclear medicine specialists, and nurses with a formal mandate of their respective Dutch national associations. It is published online (in Dutch).^18^

### Implementation

The study was carried out at Amphia Hospital (Breda, the Netherlands), an 837-bed hospital treating 380 new breast cancer cases annually. The multidisciplinary team meeting in the Amphia hospital takes place once a week. There are 5-9 new patients with breast cancer presented every week. Here, tumor board preparation is performed by nurse practitioners and consists of collection and entry of relevant patient data in a form in the EHR. During the tumor board this form is updated, and after approval its status changes from draft to final. All related clinical documentation within the EHR is performed by surgical and medical oncologist departments (ancillary departments have their own information systems).

We distinguished three different tumor boards: preoperative, postoperative, and postneoadjuvant-postoperative. For each, the standardized and structured tumor board forms were composed using the data items from the national information standard that are relevant for the different tumor board types (eg, the field “cT” [clinical tumor stage; Classification of Malignant Tumors UICC/AJCCe] was included in the preoperative form where “pT” [pathologic tumor stage; Classification of Malignant Tumours UICC/AJCC] was included in the postoperative forms; see Appendix Table A1).

For the purpose of the study, these forms were implemented in the hospital’s EHR (Epic Hyperspace, Verona, WI). EHR functionality was configured for generating full-text clinical notes from entered form data. The generated clinical notes were subsequently reused in correspondence to the patient’s GP. End users were trained to work with the standardized forms before introduction into daily clinical practice.

It should be noted that Amphia already used forms in the EHR for structured tumor board reporting at baseline. However, the previous forms were less comprehensive, not associated with any terminology system, and not aligned with the national information standard and did not generate full-text clinical notes.

Finally, a data extraction and transformation process was developed to automatically upload the information captured in the tumor board reports to the NCR database. Electronic messaging used Health Level 7–Fast Healthcare Interoperability Resources (HL7 FHIR, Ann Arbor, MI).

### Data Collection

Both draft and final tumor board reports, created as part of routine clinical care, were collected in baseline and effect measurements. Cancer registrars prepared tumor board reports in a sandbox environment (a 1-day-old copy of the production environment) of the EHR.

In addition, time required for tumor board preparation (by nurse practitioners) and tumor board discussion was measured by manually clocking each case. Before the measurements, we defined start and stop indications per task. Likewise, it was decided how to deal with potential interruptions. To check consistency in time measurements, the first batch was measured by two researchers.

Finally, a questionnaire, consisting of 25 statements regarding the usability of the tumor board forms was created. The statements were divided over the domains simplicity, clarity, readability, and general impression. Each statement was scored on a 4-point scale, with higher values indicating better usability. Questionnaire responses were obtained from the medical professionals (n = 4) who were actively involved in tumor board preparations.

### Assessing Quality of (Draft) Tumor Board Reports

The goal of a tumor board report is to reflect the outcome of the multidisciplinary case review and communicate the recommended course of action. As such, it should contain all tumor board data items substantiating the final recommendation. For an individual case, this corresponds to a set of data items (and their values) that make up a path through the decision tree(s) in question.

**FIG 1.**
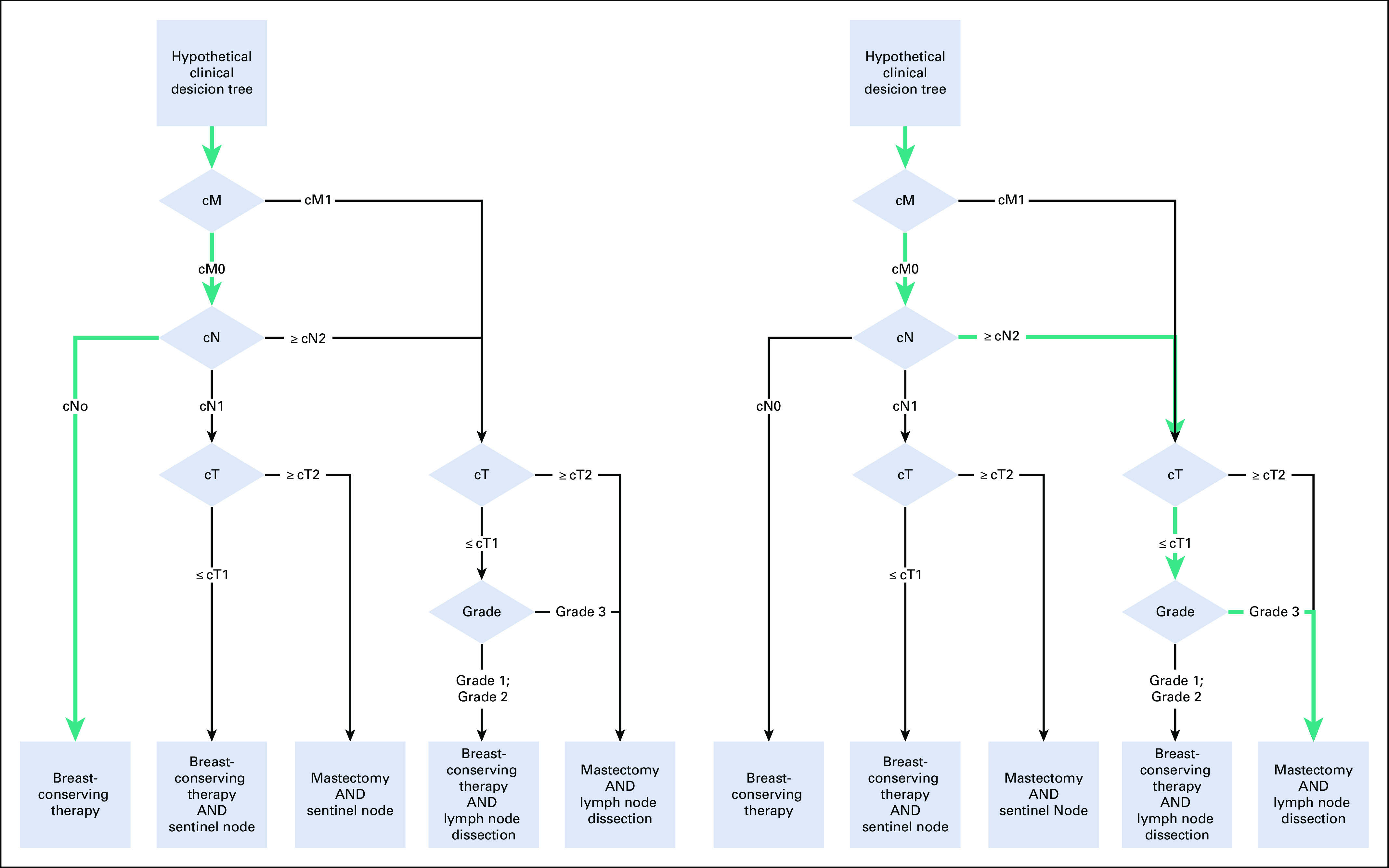
Example of a hypothetical clinical decision tree (CDT). The nodes (diamonds) represent patient and disease characteristics, the branches represent values of these characteristics, and the leaves at the bottom (rectangles) contain recommendations. Every patient runs through the CDT (top down) on a single, individual path, passing a selection of the characteristics leading to a recommendation. As indicated by the green paths, on the left panel two data items are required to be provided with a recommendation; on the right panel this is four.

This also means that the minimally required set of data varies from case to case. For example, according to the guideline for breast cancer, if a patient has metastatic disease, details about the primary tumor or lymph node involvement are irrelevant when selecting primary treatment. However, for nonmetastatic disease, these details are required. To complicate matters, there may be multiple paths in a tree leading to a single recommendation. As a result, determining which (and consequently how many) data items should have been reported for individual cases becomes difficult or even impossible in case of missing data (Fig 1).

Quality of a tumor board report was therefore operationalized as follows. Relevant subsets defined previously (*see* “Definition of the National Information Standard”) were considered the gold standard for each type of tumor board. Completeness was defined as the number of data items contained in the report divided by the number of data items in the relevant subset. Sample size calculations regarding this primary objective indicated a minimum number of 30 reports for demonstrating a statistically significant difference.

Considering it is common that only a few data items are required to complete a path through a decision tree and thus determine the appropriate guideline recommendation, completeness < 100% is expected and does not indicate a low-quality report (Fig 1). Therefore, the measure should not be used in an absolute sense. However, it can be used to measure changes (eg, in a baseline and intervention setting).

To additionally measure the impact on quality of tumor board preparation, we compared the scored data items of the tumor board reports in draft status with their final version and compared the scored data items of the drafts prepared by nurse practitioners with those prepared by cancer registrars.

### Assessing Feasibility of Data Reuse for Cancer Registries

To assess the potential for reusing tumor board data for cancer registries, we determined the overlap between the data items in the tumor board forms and the data items currently registered in the NCR for breast cancer.

### Statistical Evaluation

For all statistical analyses, two-sided unpaired *t* tests were used to compare data from before and after implementation. A *P* value < .05 was considered to be statistically significant.

## RESULTS

### Implementation

The national information standard for breast cancer was derived as described in Methods and is composed of 121 data items, 113 of which were found in the guideline. The number of data items for preoperative, postoperative, and postneoadjuvant-postoperative tumor boards forms were 37, 39, and 37, respectively (Table 1).

**TABLE 1.**
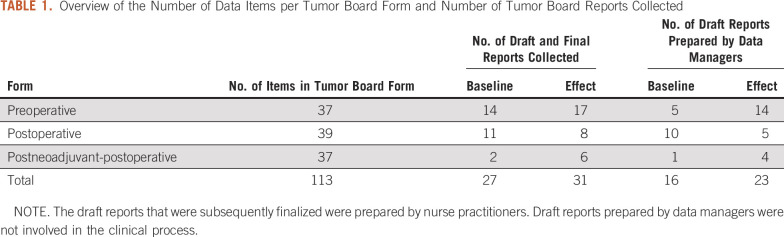
Overview of the Number of Data Items per Tumor Board Form and Number of Tumor Board Reports Collected

Standardized structured tumor board forms, automatic text generation for GP letters, and HL7 FHIR–based message exchange to the NCR were successfully implemented. The FHIR message definitions can be found online on simplifier.net.^19^

### Assessing Quality of Tumor Board Reports

#### Clinical documentation.

Draft and final tumor board reports were collected from 27 and 31 patients in baseline and effect measurements, respectively (Table 1). Measurements for every patient were performed for 5 subsequent weeks in baseline and in effect setting. Completeness of final tumor board reports increased from 39.5% in baseline to 45.4% in effect measurements (*P* = .04; Table 2).

**TABLE 2.**
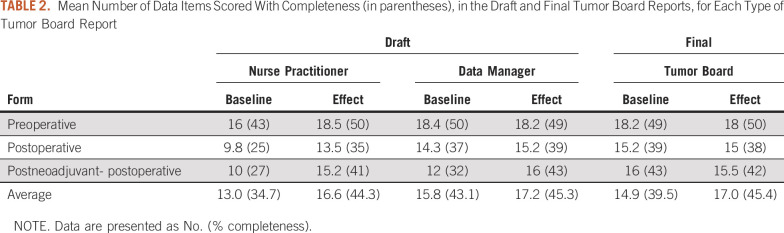
Mean Number of Data Items Scored With Completeness (in parentheses), in the Draft and Final Tumor Board Reports, for Each Type of Tumor Board Report

During the tumor board meeting in baseline on average (14.9 − 13 =) 1.9 data items were added to the report. In the effect measurements this delta was (17 − 16.6 =) 0.4 data items (Table 2). This change was not statistically significant.

At baseline, when comparing values of individual data items between the draft and final report, 26 out of 414 (6.3%) were documented with different values. In the effect measurements, different values were recorded in 22 out of 531 (4.1%) data items (Table 3). This change was not statistically significant.

**TABLE 3.**
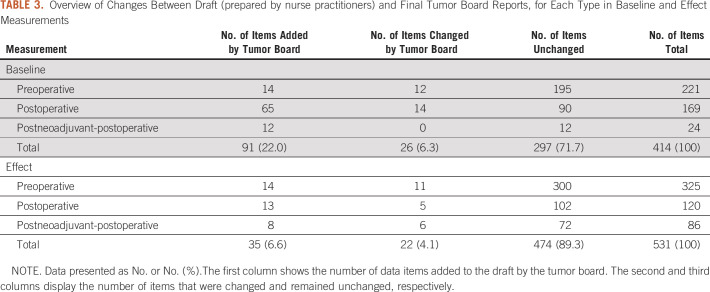
Overview of Changes Between Draft (prepared by nurse practitioners) and Final Tumor Board Reports, for Each Type in Baseline and Effect Measurements

#### Tumor board preparation by cancer registrars.

Cancer registrars prepared 16 and 23 cases in baseline and effect measurements, respectively (Table 1). When comparing draft tumor board reports prepared by nurse practitioners and cancer registrars, no statistically significant difference was found regarding completeness in baseline and effect measurements (Table 2).

In 16 (draft) tumor board reports in the baseline measurement, an absolute number of 210 data items were scored either by nurse practitioners or by cancer registrars. Out of these, 3 (1.4%) data items were recorded by nurse practitioners only and 11 (5.2%) data items by cancer registrars only. A total of 173 (82.4%) data items were recorded by both with equal values, and 23 (11.0%) were recorded with discordant values.

Similarly, in the effect measurement, 361 data items were scored across 23 (draft) tumor board reports. Here, 14 (3.9%) data items were only scored by nurse practitioners, 46 (12.7%) were only scored by cancer registrars, 286 (79.2%) data items were documented by both with corresponding values, and 15 (4.2%) data items were recorded with disagreeing values (Table 4).

**TABLE 4.**
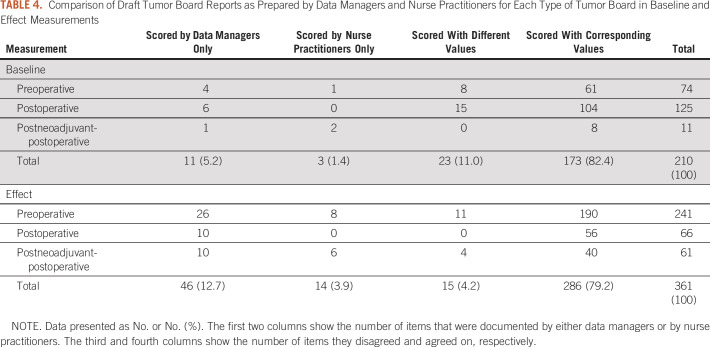
Comparison of Draft Tumor Board Reports as Prepared by Data Managers and Nurse Practitioners for Each Type of Tumor Board in Baseline and Effect Measurements

#### Workload.

Mean time involved with tumor board preparation by nurse practitioners did not change significantly (from 4:06 minutes [SD = 1:44] to 4:39 minutes [SD = 1:59]; *P* = .28). Time for tumor board discussion per patient did not change significantly (from 2:19 minutes [SD, 1:27] to 2:43 minutes [SD, 1:41]; *P* = .35; Table 5).

**TABLE 5.**
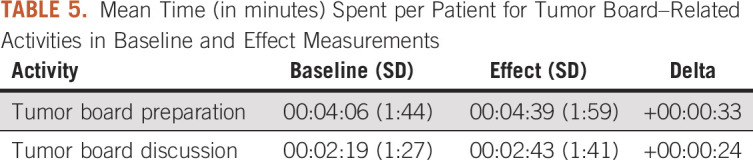
Mean Time (in minutes) Spent per Patient for Tumor Board–Related Activities in Baseline and Effect Measurements

### Assessing Feasibility of Data Reuse for Cancer Registries

Standardized tumor board reports included 50% (61/122) of the data items carried in the NCR (Appendix Table A1).

### End-User Satisfaction

The mean overall usability score (range, 1-4) was 2.63 in baseline and 2.84 in effect measurement, suggesting an overall improvement. Distributed over the subdomains, the mean scores were: simplicity, 2.75 and 2.75; clarity, 2.50 and 2.96; readability, 2.71 and 3.00; and general impression, 2.69 and 3.22 in baseline and effect measurements, respectively.

## DISCUSSION

This study shows that implementation of a national standard for tumor board reports improves the quality of clinical documentation and is possible without increasing the clinical workload. At the same time, our work enables data reuse for secondary purposes like cancer registration.

Potential downsides of structured reporting may include perceived disproportionate burden to physician workload and limitations in reporting freedom.^20^ On the basis of the results on user satisfaction, our study did not corroborate these presumed downsides.

However, as mentioned in Methods, it should be noted that Amphia already used structured EHR forms for tumor board reporting at baseline. Compared with coming from a completely free-text baseline, this could have led to underestimation of the extra effort required for structured reporting but might also have led to underestimation of the improvement in clinical documentation quality. Our results are comparable to those in pathology, where a national standard for structured reporting also improved completeness of clinically relevant data.^16^

The completion rate of approximately 50% may seem low, but it needs to be interpreted carefully. Indeed, it can be partly explained by certain data items not being filled in in clinical practice. Yet, as demonstrated by the logic tree in Figure 1, on a per-case basis this low completion does not imply that a similar number of data items required for guideline-based treatment decisions were missing.

Another reason for lower completion may reside in usability issues related to EHR systems. Indeed, there are recent studies showing a relation between physician burnout and registration burden and EHR usability.^21^ The degree to which usability can be taken into account when implementing an information standard is limited by the possibilities of the EHR system. Measures that were taken to minimize frustration were that no items were considered mandatory, taking into account that in clinical practice cases do occur where information simply is not available. Second, the forms to a degree allow hiding of information that was not required in specific cases. For example, detailed information required for a lesion is only shown to the user if a lesion is actually present.

With respect to data reuse for cancer registries, approximately 50% of data items defined in tumor board forms are currently carried in the NCR. The actual degree of reuse potential depends on the completeness of the tumor board reports. Despite the improvement in documentation quality, we observed an overall low completeness of tumor board reports. This may limit ability for data reuse in practice. Low completeness could be partially explained by not documenting negative findings (eg, not explicitly documenting “M0” in absence of metastatic disease). Lack of disciplined use of structured reporting forms by clinicians is a well-known phenomenon often attributed to poor EHR usability and the aforementioned (perceived) disproportionate burden and limitations in reporting freedom.^22^

We investigated the possibility to have cancer registrars assist in tumor board preparation. As there were no significant differences in completeness and only a limited number of discrepancies in scored data items between draft tumor board forms prepared by nurse practitioners and cancer registrars, the results hint at the possibility of using cancer registrars for this purpose. In the absence of a ground truth, we were not able to evaluate discrepancies in documentation between nurse practitioners and cancer registrars and establish which party was correct (if any). However, the number of data items scored with different values decreased from 11% in baseline to 4.1% in effect measurements.

This change was largely explained by the fact that the (introduced) standardized tumor board forms prevented scoring values that were not part of the official TNM classification system (eg, cMX). To evaluate the remaining discordant values and get a better understanding of the cancer registrar drafts, additional investigation would be recommended.

To evaluate another avenue that might reduce tumor board preparation workload, we estimated the amount of information in the tumor board form that is generated by ancillary services, like radiology and pathology (Appendix Table A1). This suggested that 59% of the data items in the forms could be automatically prepopulated if supported by the underlying technology. Actual benefits, like with reuse of data from the tumor board report, depend on the degree to which ancillary departments report required information.

Evaluation regarding usability in the effect measurement showed an improvement over the baseline, although the number of participants in the survey was low. This was partially due to the fact that only a limited number of clinicians are actively involved in the information management tasks surrounding tumor board meetings. The survey results were consistent with the positive personal feedback we received from these clinicians.

EHR implementations of structured reporting are not unique but are usually based on local physician preferences. The strength of our approach is that it is based on a national standard that is derived from the national guideline and enables evaluation of guideline adherence. As such, this study provides a road map for tumor board meetings for tumors other than breast cancer.

## Figures and Tables

**TABLE A1.**
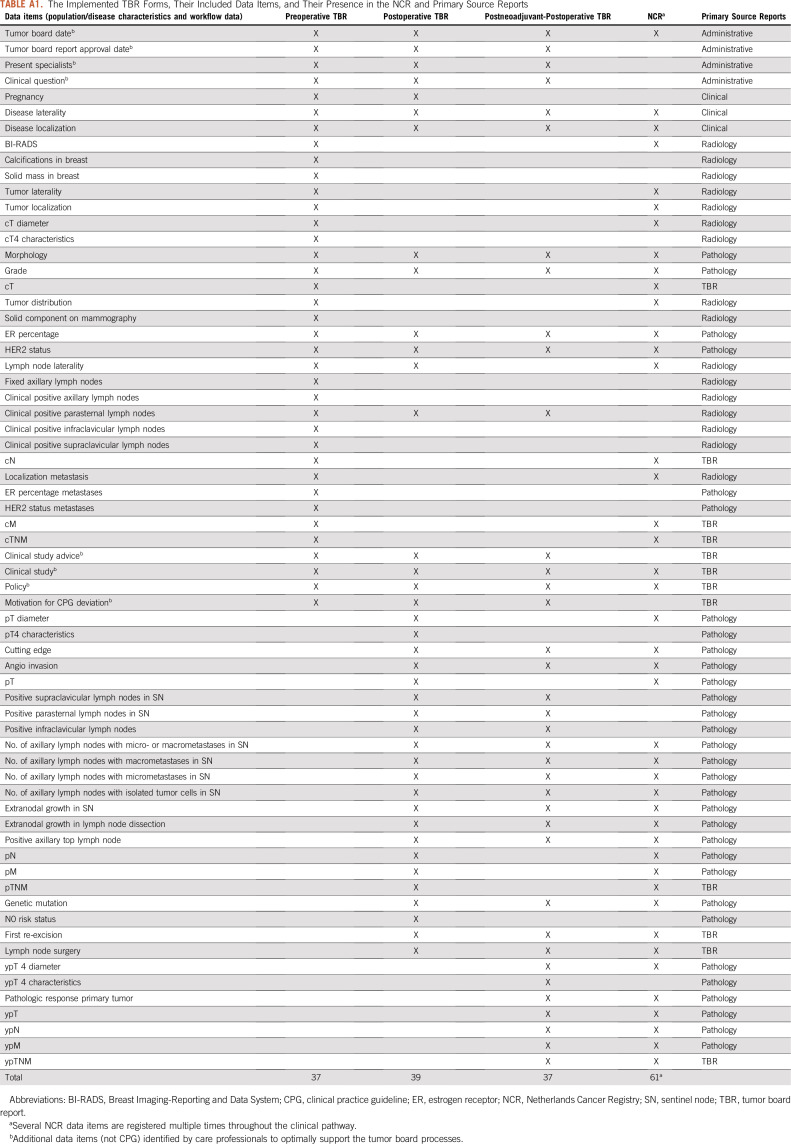
The Implemented TBR Forms, Their Included Data Items, and Their Presence in the NCR and Primary Source Reports

## APPENDIX

### Distribution of Data Items in the Information Standard

The distribution of tumor board report data items by their primary source is: administrative 12 (10.6%), clinical 12 (10.6%), radiologic 16 (14.2%), pathologic 51 (45.1%). For 22 data items (19.5%), the tumor board itself is the primary source. Standardized tumor board reports included 50% (61/122) of data items also registered retrospectively in the NCR (Appendix Table A1).
